# Reference values for 2‐dimensional and M‐mode echocardiography in Friesian and Warmblood horses

**DOI:** 10.1111/jvim.15938

**Published:** 2020-10-24

**Authors:** Ingrid Vernemmen, Lisse Vera, Glenn Van Steenkiste, Gunther van Loon, Annelies Decloedt

**Affiliations:** ^1^ Equine Cardioteam Ghent University, Department of Large Animal Internal Medicine, Faculty of Veterinary Medicine Ghent University Merelbeke Belgium

**Keywords:** aortic rupture, aortopulmonary fistula, cardiology, heart

## Abstract

**Background:**

Echocardiographic reference intervals for Friesian horses are poorly described.

**Objectives:**

To obtain reference intervals for echocardiographic measurements in Friesians and compare these with Warmbloods.

**Animals:**

One hundred healthy adult Friesians and 100 healthy adult Warmblood horses.

**Methods:**

Cross‐sectional study. Two‐dimensional and M‐mode echocardiographic images were obtained. Echocardiographic measurements, including size, area, and volumetric measurements of left atrium, left and right ventricle, aorta, and pulmonary artery, were performed. Measurements were compared between the 2 breeds using an independent samples *t* test with Bonferroni correction for multiple comparisons.

**Results:**

Reference ranges for standard echocardiographic measurements in Friesians were obtained. Several left ventricular measurements were significantly smaller in Friesians compared to Warmbloods, such as the left ventricular end‐diastolic volume using the 4‐chamber modified Simpsons' method (99.85% confidence interval for the difference [CI] = −245 to −63). Also the right ventricular end‐diastolic and peak‐systolic internal diameter were smaller in Friesians (99.85% CI = −1.33 to −0.6 and 99.85% CI = −1.54 to −0.76, respectively). Fractional shortening (99.85% CI = 0.61‐6) and ejection fraction (99.85% CI = 0.21‐4.6) were significantly larger. No structural effects of systemic hypertension, such as concentric hypertrophy, were detected.

**Conclusions and Clinical Importance:**

Our study provides reference intervals for echocardiographic measurements in Friesians useful in a clinical setting. In general, the left ventricular dimensions in Friesians were significantly smaller compared to Warmbloods, emphasizing the need for breed‐specific reference intervals.

**Abbreviations**
4C: 4‐chamber view
4C‐AL (in subscript): based on the 4‐chamber area‐length method
4C‐MOD (in subscript): based on the 4‐chamber modified Simpson's method
AoD: aortic sinotubular diameter
BW: body weight
CI: confidence interval
d_end_: end‐diastole
EF: ejection fraction
FS: fractional shortening
HR: heart rate
IVS: interventricular septal thickness
LA: left atrium
LAA: left atrial area
LAD: left atrial diameter
LV: left ventricle
LVA: left ventricular area
LVFW: left ventricular free wall thickness
LVID: left ventricular internal diameter
LVL: left ventricular length
LVOT: left ventricular outflow tract view
LVV: left ventricular volume
max (in subscript): at maximal dimension
MWT: mean wall thickness
PAD: pulmonary artery diameter
RV: right ventricle
RVID: right ventricular diameter
RWT: relative wall thickness
s_end_: end‐systole
s_peak_: peak systole
SV: stroke volume

## 1 INTRODUCTION

Echocardiography is an essential part of the structural and functional evaluation of the equine heart. Standardized echocardiographic images and measurements are established,^1^, ^2^, ^3^, ^4^ as is their relation with age, sex, growth, training, body weight (BW), and breed.^5^, ^6^, ^7^, ^8^, ^9^ Breed has an important influence on echocardiographic measurements in horses,^5^ dogs^10^ and humans.^11^ Echocardiographic reference intervals are available for Thoroughbreds,^2^, ^12^ Standardbreds,^13^, ^14^ Warmbloods,^6^, ^15^, ^16^ Arabian horses,^17^ Icelandic horses,^9^ and ponies.^18^ There is limited information on reference intervals for Friesian horses.^19^

The Friesian breed is marked by considerable inbreeding, which presumably has contributed to their distinctive conformation and higher prevalence of several diseases.^20^, ^21^ Friesians are predisposed to aortic rupture near the ligamentum arteriosum in conjunction with aortopulmonary fistulation. The condition can occur as an acute event of sudden death, or can be more chronic with less specific clinical signs, making the diagnosis challenging. Echocardiography is essential in the diagnostic protocol, using conventional and nonconventional views to identify the lesion and assess the consequences on the heart's structure and function.^22^, ^23^, ^24^ In order to be able to comprehensively and objectively investigate cardiac disorders and evaluate their effect on cardiac function in Friesians, reference intervals are needed to distinguish normal from the abnormal.^5^, ^13^

Friesians react differently with training^25^ and reach their anaerobic threshold at lower workloads than other horse breeds.^26^ We therefore hypothesized that the cardiac diameters, areas, and volumes would be smaller in the Friesians compared to the Warmbloods. Because of the higher systemic blood pressure and higher arterial wall stiffness in Friesians,^27^ we also hypothesized that left ventricular concentric hypertrophy^28^ would be detected in Friesians but not Warmblood horses. The goal of our study was to determine reference intervals for standard echocardiographic variables for the Friesian breed and to compare these with measurements in Warmblood horses.

## 2 MATERIALS AND METHODS

### 2.1 Animals

A cross‐sectional study was performed. The study sample consisted of 100 healthy adult Friesians aged (median [range]) 10 [3‐26] years and 100 healthy adult Warmbloods aged 8 [3‐25] years. The Friesians consisted of 72 mares, 23 geldings, and 5 stallions and the Warmbloods of 61 mares, 35 geldings, and 4 stallions. Whether the horse was in training, as opposed to at rest in the field, at the time of the study (yes/no) was registered, as well as information about pregnancy and lactation. Mares over 6 months of gestation or within 1 week after parturition were excluded. In all horses, auscultation, echocardiography, and ECG were performed. Inclusion criteria were: no more than a 2/6 left‐ or right‐sided systolic or diastolic murmur on auscultation, no more than mild valvular regurgitation visible with Doppler ultrasound examination, and absence of atrial and ventricular premature beats during a 15 minutes ECG recording at rest. Height at the withers, body length, and chest circumference were measured for every horse. Body length was determined from the most cranial point of the shoulder to the tuber ischiadicum, chest circumference was measured right behind the elbow. Body weight was estimated using the following formula^29^, ^30^:

$$\mathrm{BW}=\frac{{\text{chest circumference}}^{2}*\text{body length}}{11900}$$

### 2.2 Echocardiography

Echocardiography was performed in the standing, nonsedated horse (Vivid IQ, GE Healthcare, Diegem, Belgium). A 1.5 to 4.6 MHz phased array probe (M5Sc‐RS) was used at a frequency of 1.7/3.3 MHz with simultaneous ECG recording (base‐apex). Two‐dimensional images included the right‐parasternal long‐axis 4‐chamber view (4C) and the right‐parasternal long‐axis view of the left ventricular outflow tract (LVOT). M‐mode images were obtained of the right‐parasternal short‐axis view of the left ventricle (LV) at the chordal level.^31^ Images were stored for off‐line analysis.

### 2.3 Off‐line analysis

All images were blinded for breed and analyzed by a single observer, using dedicated software (EchoPAC Software Version 203, GE Healthcare, Diegem, Belgium). The mean of 3 consecutive cycles was used for further analysis. Cycles following a second‐degree atrioventricular or sinoatrial block were not used for analysis, and resulted in analysis of 3 nonconsecutive cycles in some cases. Three nonconsecutive cycles were also used when image quality was inadequate to obtain 3 consecutive cycles. Heart rate (HR) was determined as the mean of 3 RR intervals of the 3 consecutive cycles on the 4C view. Images were only recorded and analyzed if HR was below 50 beats/min. The measurements on the 4C view included the left atrial (LA) diameter at the mitral valve annulus and at maximal dimension parallel to the mitral valve annulus at end‐systole and end‐diastole (LADs_end_, LAD_max_s_end_, LADd_end_, LAD_max_d_end_), the LA area at end‐systole and end‐diastole (LAAs_end_ and LAAd_end_),^4^ and the LV area and length at end‐systole and end‐diastole (LVAs_end_, LVLs_end_, LVAd_end_, LVLd_end_).^31^ The end‐systolic and end‐diastolic measurements were timed 1 frame before mitral valve opening and 1 frame after mitral valve closure, respectively. On the LVOT view, the aortic diameter at the level of the sinotubular junction and the pulmonary artery diameter in short‐axis were measured at peak systole and end‐diastole (AoDs_peak_, AoDd_end_, PADs_peak_, and PADd_end_)^31^, for which the peak systolic measurement was performed at maximal aortic valve opening and the end‐diastolic measurement 1 frame before aortic valve opening. The ratio of the pulmonary artery diameter and aortic diameter at peak systole was calculated (PADs_peak_/AoDs_peak_). The M‐mode variables, measured at the chordal level, included the right ventricular (RV) internal diameter at peak systole and end‐diastole (RVIDs_peak_ and RVIDd_end_), the interventricular septal thickness at peak systole and end‐diastole (IVSs_peak_ and IVSd_end_), the LV internal diameter at peak systole and end‐diastole (LVIDs_peak_ and LVIDd_end_), and the LV free wall thickness at peak systole and end‐diastole (LVFWs_peak_ and LVFWd_end_).^31^ Peak systolic measurements were performed at maximal contraction and end‐diastolic measurements at maximal ventricular filling. Fractional shortening (FS), relative wall thickness of the LV (RWT), and mean wall thickness of the LV at end‐diastole (MWTd_end_) were calculated using the following formulas^31^:

$$\mathrm{FS}=\frac{{\text{LVIDd}}_{\mathrm{end}}-{\text{LVIDs}}_{\text{peak}}}{{\text{LVIDd}}_{\mathrm{end}}}*100$$

$$\mathrm{RWT}=\frac{{\text{IVSd}}_{\mathrm{end}}+{\text{LVFWd}}_{\mathrm{end}}}{{\text{LVIDd}}_{\mathrm{end}}}$$

$${\text{MWTd}}_{\mathrm{end}}=\frac{{\text{IVSd}}_{\mathrm{end}}+{\text{LVFWd}}_{\mathrm{end}}}{2}$$

Volumetric measurements of the LV (LVV) were performed with built‐in algorithms using 2 different methods: (a) the 4C area‐length method and (b) the 4C modified Simpson's method, both based on the measurements performed on the 4C view.^32^, ^33^ Based on the 4C area‐length method, the LVV is calculated as:

$${\mathrm{LVV}}_{4C-\mathrm{AL}}=0.85*\frac{{\mathrm{LVA}}^{2}}{\mathrm{LVL}}$$

Using the 4C modified Simpson's method, the LVV is calculated as:

$${\mathrm{LVV}}_{4C-\mathrm{MOD}}=\frac{\pi }{4}\;\Sigma \left(\frac{a_{i}^{2}}{n}\right)*\mathrm{LVL},\text{with}\;a_{i}=\text{diameter of}\;i^{\mathrm{th}}\;\text{disk and}\;n=16$$

The LVV was determined at end‐systole and end‐diastole using each method (LVV_4C‐AL_s_end_, LVV_4C‐AL_d_end_, LVV_4C‐MOD_s_end_, LVV_4C‐MOD_d_end_). The stroke volume was calculated as the difference of the end‐diastolic and end‐systolic volume (SV_4C‐AL_, SV_4C‐MOD_), and the ejection fraction as the ratio of the stroke volume over the corresponding end‐diastolic volume (EF_4C‐AL_, EF_4C‐MOD_).

### 2.4 Data analysis

Data analysis was performed using dedicated software (SPSS Statistics 26, IBM, Brussels, Belgium). Variables were tested for normality by analyzing the Q‐Q plots. Normally distributed data are presented as mean ± SD and nonparametric data as median [range]. Categorical data are presented as proportions. The homogeneity of variances was tested with the Levene's test. Age was compared between the 2 breeds using a Mann‐Whitney *U* test and height at the withers, chest circumference, body length, estimated BW, and HR were compared between breeds using an independent sample *t* test. Sex distribution between the 2 breeds was compared using a Fisher's Exact test. Training status, pregnancy, and lactation were compared using a Chi‐square test. For each variable, 95% reference ranges were constructed and 90% confidence intervals (CIs) were calculated for the lower and upper limits,^34^, ^35^ using dedicated software based on standard methods (Reference Value advisor v2.1, National Veterinary School, Toulouse, France). No transformations for normality were performed. If outliers were present, their influence on the reference ranges was assessed and if found minimal, the outliers were retained in the database. Independent sample *t* tests were performed for each variable and Bonferroni correction was performed to correct for multiple comparisons. The overall significance level was *P* < .05. For each variable, *P* values were considered significant if *P* < .002 (= 0.05/34 variables) and 99.85% CI for the mean difference were calculated.

## 3 RESULTS

Summary statistics of the study sample are given in Table 1. Five Friesians and 1 Warmblood horse were excluded from the study due to stress‐induced high HR (>50 beats/min). The aortic diameter was not measurable in 3 Friesians and the pulmonary artery diameter in 1 Friesian because of inadequate image quality. Training status was unknown in 13 Warmbloods, pregnancy and lactation status was unknown in 10 Warmbloods, and age was unknown in 1 Warmblood horse. The height at the withers was significantly lower in Friesians compared to Warmbloods (Friesians 162 ± 4 cm, Warmbloods 167 ± 5 cm; *P* < .001) while the chest circumference (Friesians 199 ± 7 cm, Warmbloods 193 ± 8 cm; *P* < .001) and body length (Friesians 188 ± 10 cm, Warmbloods 179 ± 9 cm; *P* < .001) were significantly larger in Friesians. Friesians also had a significantly higher estimated BW (Friesians 627 ± 59 kg, Warmbloods 561 ± 63 kg; *P* < .001). Significantly more Friesians were in training (*P* < .001) or pregnant (*P* = .006). No difference could be detected between the breeds for age (*P* = .29), HR (*P* = .43), sex (*P* = .24), or lactation (*P* = .07).

**TABLE 1:** Summary statistics of study sample

Variables	Friesians (N = 95)			Warmbloods (N = 99)			*P* value	95% CI for the difference
Estimated BW (kg)	627 ± 59			561 ± 63			**<.001**	**(49‐83)**
Height at the withers (cm)	162 ± 4			167 ± 5			**<.001**	**(−7 to −4)**
Chest circumference (cm)	199 ± 7			193 ± 8			**<.001**	**(4‐8)**
Body length (cm)	188 ± 10			179 ± 9			**<.001**	**(7‐12)**
Heart rate (bpm)	39 ± 5			38 ± 6			.43	(−1 to 2)
Age (y)^a^	10 [3‐26]			8 [3‐25]			.29	
Sex	*Mare*	*Gelding*	*Stallion*	*Mare*	*Gelding*	*Stallion*		
	68	22	5	61	34	4	.24	
Training^b^	*Yes*	*No*		*Yes*	*No*			
	90	5		62	24		**<.001**	
Pregnancy^c^	*Yes*	*No*	*NA*	*Yes*	*No*	*NA*		
	13	55	27	2	49	38	**.006**	
Lactation^c^	*Yes*	*No*	*NA*	*Yes*	*No*	*NA*		
	8	60	27	3	48	38	.071	

*Note*: Data are presented as numbers, as mean ± SD or as median [range]. Significance level was *P* < .05. Significant *P* values and 95% CI are marked in bold.

Abbreviations: 95% CI, 95% confidence interval; bpm, beats per minute; BW, body weight; N, number of horses; NA, not applicable (male).

^a^ Based on 95 Friesians and 98 Warmblood horses, age was not recorded in 1 Warmblood horse.

^b^ Based on 95 Friesians and 86 Warmblood horses, training status was not recorded in 13 Warmblood horses.

^c^ Based on 95 Friesians and 89 Warmblood horses, pregnancy and lactation status was not recorded in 10 Warmblood mares.

The echocardiographic measurements and their reference intervals are displayed in Tables 2 and 3. All LV and RV dimensions were significantly smaller in Friesians compared to Warmbloods, except for the LVIDd_end_. Of the LA dimensions, only the LADs_end_ was significantly smaller in Friesians compared to Warmbloods. Apart from a smaller AoDs_peak_, the dimensions of the aorta and pulmonary artery were not significantly different between the 2 breeds. The IVSd_end_ was significantly smaller in Friesians, on the other hand, the IVSs_peak_ was significantly larger in Friesians. The functional indices FS and the EF_4C‐MOD_ were significantly higher in the Friesians compared to the Warmbloods. No significant difference could be found for the EF_4C‐AL_.

**TABLE 2:** Results of 2‐dimensional echocardiographic measurements for Friesians and Warmbloods

Variables	Friesians				Warmbloods				Mean difference ± SD	*P* value (99.85% CI) for the difference
	N	Mean ± SD	Lower limit of the reference interval (90% CI)	Upper limit of the reference interval (90% CI)	N	Mean ± SD	Lower limit of the reference interval (90% CI)	Upper limit of the reference interval (90% CI)		
LADd_end_ (cm)	95	9.4 ± 0.87	7.7 (7.5‐7.9)	11.2 (10.9‐11.4)	99	9.5 ± 0.90	7.7 (7.5‐8.0)	11.3 (11.1‐11.6)	−0.12 ± 0.127	.35 (−0.53 to 0.29)
LADs_end_ (cm)	95	9.8 ± 0.68	8.4 (8.3‐8.6)	11.1 (10.9‐11.3)	99	10.1 ± 0.74	8.6 (8.5‐8.8)	11.6 (11.4‐11.8)	−0.34 ± 0.102	**.001 (−0.67 to −0.016)**
LAD_max_d_end_ (cm)	95	10.3 ± 0.96	8.4 (8.1‐8.7)	12.2 (11.9‐12.5)	99	10.3 ± 1.00	8.3 (8.0‐8.5)	12.3 (12.0‐12.5)	0.04 ± 0.14	.75 (−0.41 to 0.50)
LAD_max_s_end_ (cm)	95	12.0 ± 0.78	10.4 (10.2‐10.6)	13.5 (13.3‐13.7)	99	11.8 ± 0.89	10.0 (9.8‐10.3)	13.6 (13.3‐13.8)	0.14 ± 0.121	.24 (−0.25 to 0.53)
LAAd_end_ (cm^2^)	95	54 ± 8.7	36 (33‐38)	71 (68‐74)	99	55 ± 9.7	36 (34‐39)	75 (72‐77)	−1.3 ± 1.32	.32 (−5.6 to 2.9)
LAAs_end_ (cm^2^)	95	87 ± 9.2	69 (67‐72)	106 (103‐108)	99	89 ± 13.5	62 (58‐66)	116 (112‐119)	−1.01 ± 1.66	.52 (−6.4 to 4.3)
LVLd_end_ (cm)	95	15.9 ± 0.95	14.0 (13.7‐14.3)	17.8 (17.5‐18.1)	99	17.0 ± 1.03	14.9 (14.7‐15.2)	19.0 (18.7‐19.3)	−1.09 ± 0.142	**<.001 (−1.5 to −0.63)**
LVLs_end_ (cm)	95	10.8 ± 0.98	8.9 (8.6‐9.2)	12.8 (12.5‐13.1)	99	11.6 ± 1.22	9.2 (8.9‐9.5)	14.0 (13.7‐14.4)	−0.79 ± 0.159	**<.001 (−1.3 to −0.25)**
LVAd_end_ (cm^2^)	95	139 ± 14.9	109 (105‐113)	169 (164‐172)	99	154 ± 19	117 (112‐122)	192 (187‐197)	−15.2 ± 2.44	**<.001 (−23 to −7.4)**
LVAs_end_ (cm^2^)	95	52 ± 8.0	36 (34‐38)	68 (66‐70)	99	60 ± 11.0	39 (36‐42)	82 (79‐85)	−8.6 ± 1.38	**<.001 (−13 to −4.2)**
LVV_4C‐AL_d_end_ (cm^3^)	95	1037 ± 178	681 (626‐735)	1392 (1319‐1468)	99	1196 ± 233	731 (671‐793)	1660 (1594‐1723)	−159 ± 29.7	**<.001 (−255 to −63)**
LVV_4C‐AL_s_end_ (cm^3^)	95	214 ± 56	103 (88‐118)	325 (309‐341)	99	272 ± 78	107 (81‐131)	419 (392‐446)	−58 ± 9.7	**<.001 (−89 to −27)**
LVV_4C‐MOD_d_end_ (cm^3^)	95	1001 ± 170	662 (607‐715)	1340 (1270‐1417)	99	1155 ± 221	714 (657‐774)	1596 (1533‐1656)	−154 ± 28.2	**<.001 (−245 to −63)**
LVV_4C‐MOD_s_end_ (cm^3^)	95	226 ± 55	116 (102‐131)	335 (320‐351)	99	290 ± 75	140 (120‐160)	440 (419‐461)	−64 ± 9.4	**<.001 (−95 to −34)**
SV_4C‐AL_ (cm^3^)	95	822 ± 159	510 (468‐553)	1136 (1090‐1179)	99	924 ± 185	554 (507‐604)	1293 (1241‐1344)	−101 ± 24.7	**<.001 (−181 to −22)**
SV_4C‐MOD_ (cm^3^)	95	776 ± 151	474 (434‐516)	1077 (1033‐1119)	99	865 ± 175	516 (471‐563)	1215 (1165‐1262)	−89 ± 23.5	**<.001 (−166 to −14)**
EF_4C‐AL_ (%)	95	79 ± 4.5	70 (69‐72)	88 (87‐89)	99	77 ± 4.5	68 (67‐70)	86 (85‐87)	1.9 ± 0.65	.003 (−0.15 to 4.0)
EF_4C‐MOD_ (%)	95	77 ± 4.9	67 (66‐69)	87 (86‐88)	99	75 ± 4.6	66 (65‐67)	84 (83‐85)	2.4 ± 0.68	**.001 (0.21‐4.6)**
AoDd_end_ (cm)	92	5.9 ± 0.48	5.0 (4.8‐5.1)	6.9 (6.8‐7.0)	99	6.1 ± 0.55	5.0 (4.9‐5.2)	7.2 (7.1‐7.4)	−0.20 ± 0.075	.008 (−0.44 to 0.041)
AoDs_peak_ (cm)	92	6.5 ± 0.45	5.6 (5.5‐5.7)	7.4 (7.3‐7.5)	99	6.8 ± 0.58	5.6 (5.5‐5.8)	7.9 (7.8‐8.1)	−0.27 ± 0.075	**<.001 (−0.51 to −0.025)**
PADd_end_ (cm)	94	4.9 ± 0.42	4.0 (3.9‐4.1)	5.7 (5.6‐5.8)	99	5.1 ± 0.51	4.0 (3.9‐4.2)	6.1 (5.9‐6.2)	−0.19 ± 0.067	.005 (−0.41 to 0.026)
PADs_peak_ (cm)	94	5.2 ± 0.41	4.4 (4.3‐4.5)	6.0 (5.9‐6.1)	99	5.3 ± 0.48	4.4 (4.2‐4.5)	6.3 (6.1‐6.4)	−0.12 ± 0.064	.07 (−0.32 to 0.09)
PADs_peak_/AoDs_peak_	91	0.80 ± 0.072	0.66 (0.64‐0.68)	0.95 (0.92‐0.97)	99	0.79 ± 0.067	0.65 (0.63‐0.67)	0.92 (0.90‐0.94)	0.014 ± 0.0101	.18 (−0.019 to 0.046)

*Note*: Significance level was *P* < .002 (after Bonferroni correction). Significant *P* values and 99.85% CI are marked in bold.

Abbreviations: AoDd_end_, aortic diameter at end‐diastole; AoDs_peak_, aortic diameter at peak‐systole; CI, confidence interval; EF_4C‐AL_, ejection fraction based on 4‐chamber area‐length method; EF_4C‐MOD_, ejection fraction based on Modified Simpson's method; LAAd_end_, left atrial area at end‐diastole; LAAs_end_, left atrial area at end‐systole; LADd_end_, left atrial diameter at end‐diastole; LAD_max_d_end_, maximal atrial diameter at end‐diastole; LAD_max_s_end_, maximal atrial diameter at end‐systole; LADs_end_, left atrial diameter at end‐systole; LVAd_end_, left ventricular area at end‐diastole; LVAs_end_, left ventricular area at end‐systole; LVLd_end_, left ventricular length at end‐diastole; LVLs_end_, left ventricular length at end‐systole; LVV_4C‐AL_d_end_, left ventricular volume using the 4‐chamber area‐length method at end‐diastole; LVV_4C‐AL_s_end_, left ventricular volume using the 4‐chamber area‐length method at end‐systole; LVV_4C‐MOD_d_end_, left ventricular volume using the Modified Simpson's method at end‐diastole; LVV_4C‐MOD_s_end_, left ventricular volume using the Modified Simpson's method at end‐systole; N, number of horses; PADd_end_, pulmonary artery diameter at end‐diastole; PADs_peak_, pulmonary artery diameter at peak‐systole; SV_4C‐AL_, stroke volume based on 4‐chamber area‐length method; SV_4C‐MOD_, stroke volume based on Modified Simpson's method.

**TABLE 3:** Results of M‐mode echocardiographic measurements for Friesians and Warmbloods

Variables	Friesians				Warmbloods				Mean difference ± SD	*P* value (99.85% CI) for the difference
	N	Mean ± SD	Lower limit of the reference interval (90% CI)	Upper limit of the reference interval (90% CI)	N	Mean ± SD	Lower limit of the reference interval (90% CI)	Upper limit of the reference interval (90% CI)		
LVIDd_end_ (cm)	95	11.1 ± 0.76	9.5 (9.3‐9.8)	12.6 (12.3‐12.8)	99	11.1 ± 0.88	9.4 (9.1‐9.6)	12.9 (12.6‐13.1)	−0.06 ± 0.118	.62 (−0.44 to 0.32)
LVIDs_peak_ (cm)	95	6.3 ± 0.73	4.8 (4.6‐5.0)	7.8 (7.5‐8.0)	99	6.7 ± 0.96	4.8 (4.5‐5.0)	8.6 (8.3‐8.9)	−0.41 ± 0.122	**.001 (−0.81 to −0.019)**
RVIDd_end_ (cm)	95	2.9 ± 0.78	1.4 (1.2‐1.6)	4.5 (4.3‐4.7)	99	3.9 ± 0.82	2.3 (2.1‐2.5)	5.5 (5.3‐5.7)	−0.97 ± 0.114	**<.001 (−1.33 to −0.6)**
RVIDs_peak_ (cm)	95	2.1 ± 0.88	0.4 (0.2‐0.6)	3.9 (3.6‐4.1)	99	3.3 ± 0.81	1.7 (1.5‐1.9)	4.9 (4.7‐5.1)	−1.15 ± 0.121	**<.001 (−1.54 to −0.76)**
IVSd_end_ (cm)	95	2.8 ± 0.30	2.2 (2.2‐2.3)	3.4 (3.4‐3.5)	99	3.1 ± 0.34	2.4 (2.3‐2.5)	3.7 (3.6‐3.8)	−0.23 ± 0.046	**<.001 (−0.38 to −0.082)**
IVSs_peak_ (cm)	95	4.6 ± 0.43	3.7 (3.6‐3.8)	5.4 (5.3‐5.5)	99	4.3 ± 0.47	3.4 (3.3‐3.5)	5.3 (5.1‐5.4)	0.23 ± 0.065	**.001 (0.019‐0.44)**
LVFWd_end_ (cm)	95	2.5 ± 0.31	1.9 (1.8‐2.0)	3.1 (3.0‐3.2)	99	2.4 ± 0.31	1.8 (1.7‐1.8)	3.0 (2.9‐3.1)	0.12 ± 0.044	.005 (−0.018 to 0.27)
LVFWs_peak_ (cm)	95	4.3 ± 0.47	3.3 (3.2‐3.5)	5.2 (5.1‐5.4)	99	4.1 ± 0.49	3.2 (3.0‐3.3)	5.1 (5.0‐5.2)	0.17 ± 0.069	.02 (−0.054 to 0.39)
MWTd_end_ (cm)	95	2.67 ± 0.226	2.21 (2.16‐2.28)	3.12 (3.05‐3.18)	99	2.71 ± 0.260	2.20 (2.13‐2.27)	3.24 (3.16‐3.31)	−0.50 ± 0.035	.13 (−0.17 to 0.06)
RWT	95	0.48 ± 0.050	0.39 (0.37‐0.41)	0.59 (0.57‐0.60)	99	0.49 ± 0.061	0.37 (0.35‐0.39)	0.62 (0.60–0.63)	−0.008 ± 0.0081	.31 (−0.034 to 0.018)
FS (%)	95	43 ± 5.3	32 (30‐34)	53 (51‐55)	99	40 ± 6.3	27 (26‐29)	53 (51‐54)	3.3 ± 0.084	**<.001 (0.61‐6)**

*Note*: Significance level was *P* < .002 (after Bonferroni correction). Significant *P* values and 99.85% CI are marked in bold.

Abbreviations: CI, confidence interval; FS, fractional shortening; IVSd_end_, interventricular septal thickness at end‐diastole; IVSs_peak_, interventricular septal thickness at peak‐systole; LVFWd_end_, left ventricular free wall thickness at end‐diastole; LVFWs_peak_, left ventricular free wall thickness at peak‐systole; LVIDd_end_, left ventricular internal diameter at end‐diastole; LVIDs_peak_, left ventricular internal diameter at peak‐systole; MWTd_end_, mean wall thickness at end‐diastole; N, number of horses; RVIDd_end_, right ventricular internal diameter at end‐diastole; RVIDs_peak_, right ventricular internal diameter at peak‐systole; RWT, relative wall thickness.

## 4 DISCUSSION

In our study, echocardiographic dimensions between Friesians and Warmbloods of similar age and sex were compared. This analysis demonstrated that in general the LV and the right ventricular internal diameter of a Friesian were smaller compared to that of a Warmblood and that the functional indices were generally higher in Friesians. Next to breed, other factors might have influenced these differences, such as training level, body conformation, and pregnancy.

The higher FS and the EF_4C‐MOD_ might be explained by the fact that a smaller LV in a body of approximately the same size needs to increase its systolic function to preserve sufficient stroke volume.^36^ The right ventricular internal diameter was smaller in Friesians, which is of clinical importance as aorto‐pulmonary fistulation can induce dilatation of the right heart.^23^ The lower IVSd_end_ and higher IVSs_peak_ in Friesians, despite a similar longitudinal systolic shortening of the LV (data not presented), might be explained by the shorter LV in Friesians which could affect myocardial fiber orientation, left ventricular systolic shape, or M‐mode cursor positioning. For some variables, the differences might be accounted for by the expected between day and within observer variability for echocardiographic measurements. Nevertheless, several clinically relevant differences, namely the LV size, the functional indices, and the right ventricular internal diameter, were found, emphasizing that breed influences should be taken into account when interpreting echocardiographic measurements.

The influence of breed might be difficult to distinguish from effects of sport discipline and training level.^5^ Warmbloods are generally used in more demanding sport disciplines, such as show jumping and eventing, in comparison with Friesians. Therefore, different training levels might contribute to the smaller LV size and right ventricular internal diameter of the Friesians as well.^7^ Training induces cardiac adaptive responses such as increased LVIDd_end_, LV mass, RWT and MWTd_end_, and decreased ejection fraction and FS.^7^, ^37^ In our study sample, more Friesians were reported as being “in training” than Warmblood horses, but training level, training history, or performance were not specified. We can therefore make no definite conclusions on the effect of training and fitness in our study. Friesians have a different response to training and reach their anaerobic threshold at lower workloads than other breeds.^25^, ^26^ The anaerobic threshold is a commonly used variable to optimize training and evaluate fitness level in humans and horses.^38^ Another variable correlated with fitness and performance in horses, the maximal oxygen uptake ($V_{O_{2}}$_max_), is linked to LV size.^39^ It is therefore plausible that the different training response in Friesians is linked to a smaller LV size and right ventricular internal diameter compared to breeds that are adapted to a higher level of performance.

The significantly lower height at the withers of Friesians might also explain the smaller LV size and right ventricular internal diameter apart from breed‐specific influences. However, height at the withers is less correlated to echocardiographic measurements than chest circumference,^40^ which was significantly higher in Friesians. Body weight is correlated with echocardiographic dimensions but only the estimated BW was available in our study, using a formula validated for a variety of horse breeds but not for the Friesians.^30^ As Friesians are characterized by a different body conformation compared to Warmbloods, the estimated BW could be less reliable in this context.

In humans, changes in LV geometry are a predictor of systemic hypertension as well as a consequence of systemic hypertension.^41^ Compared to Warmbloods, Friesians have a higher systemic blood pressure.^27^ Systemic hypertension causes aortic dissection in humans, as it reduces the blood flow in the vasa vasorum causing ischemia and degeneration of the external media, thereby reducing the elasticity of the aorta.^42^ In addition, increased blood pressures cause shear stress in the aorta, creating an entry site for aortic dissection.^42^ A similar mechanism might play a role in aortic rupture in Friesians, as medial necrosis has been described in histopathological studies of Friesians with aortic rupture.^43^ Our study was conducted on the same sample of Friesians as the study in which the higher systemic blood pressure was demonstrated^27^; however, no impact of a higher systemic blood pressure on the cardiac dimensions or function, such as concentric hypertrophy,^28^, ^44^ was demonstrated.

More Friesians in our study were pregnant. Pregnancy induces several hemodynamic and cardiovascular changes in humans, such as an increased HR, increased cardiac output, mild general cardiac dilatation, increased LV and RV mass, and increased LV wall thickness.^45^ Pregnant mares are thought to undergo similar physiological adaptations such as changes in HR,^46^ the renin‐angiotensin‐aldosterone system,^47^ and biochemical blood components.^48^ Changes in echocardiographic variables in pregnant mares have not been reported. Nevertheless, although significantly more Friesians were pregnant in our study, their LV and right ventricular internal diameter were still significantly smaller. Further research is needed to investigate the influence of pregnancy,^49^, ^50^ not only the stage but also multiparity,^51^, ^52^ and lactation^53^ on echocardiographic measurements, to evaluate their interplay with other factors such as training and to determine the reversibility of these changes.

A limitation of our study was the quality of the echocardiographic images. Due to the body conformation of Friesian horses, echocardiography can be challenging.^22^ Image quality in some Friesians might have been less compared to the Warmbloods and might have influenced results. Measurements were performed by 1 blinded observer. The resulting reference intervals might be prone to interobserver variability, although this is low for standard 2‐dimensional and M‐mode echocardiographic measurements.^4^, ^13^ Accurately measured BW was not available in our study and therefore did not allow allometric scaling. However, the primary purpose of our data is to provide reference intervals, applicable in daily practice where body confirmation data might be more readily available than BW. While our data were collected from multiple breeders and genetically unrelated horses, body conformation data such as height, chest circumference, and body length showed only limited variation, suggesting that obtained intervals are a valid representation for the general Friesian population.

## CONFLICT OF INTEREST DECLARATION

Authors declare no conflict of interest.

## OFF‐LABEL ANTIMICROBIAL USE DECLARATION

Authors declare no off‐label use of antimicrobials.

## INSTITUTIONAL ANIMAL CARE AND USE COMMITTEE (IACUC) OR OTHER APPROVAL DECLARATION

The current study (EC 2018/11) was performed following the guidelines of the Ethical Committee of the Faculty of Veterinary Medicine, Ghent University, Belgium.

## HUMAN ETHICS APPROVAL DECLARATION

Authors declare human ethics approval was not needed for this study.
